# On the Nature and Treatment of Stomach and Urinary Diseases; Being an Inquiry into the Connexion of Diabetes, Calculus, and Other Affections of the Kidney and Bladder with Indigestion

**Published:** 1841-04

**Authors:** 


### Art. III.

*On the Nature and Treatment of Stomach and Urinary Diseases; being an Inquiry into the Connexion of Diabetes, Calculus, and other affections of the Kidney and Bladder with Indigestion.* By William Prout, m.d. f.r.s. &c. *Third Edition.—London,* 1840. 8vo, pp. 594.

We felt very considerable anxiety to become acquainted with this new version of a treatise which has long held a prominent place on the shelves of medical libraries.

The work is rewritten, and in size it greatly exceeds its predecessors; but its materials are arranged on the principles connected with Dr. Prout's name. We shall not limit our notice to any actual novelty the present edition of the treatise may contain, but endeavour by giving as full a statement as we can of its leading doctrines and their applications, whether now first broached or already made public, to enable our readers to form a just estimate of its scope and merits.

The volume opens with an introduction, comprising an outline of the general physiology and pathology of assimilation, and of the secretion of the bile and urine. Having stated his classification of alimentary products into the aqueous, the saccharine, the albuminous, and the oleaginous, Dr. Prout proceeds to offer some general remarks on each of these. Saccharine bodies, he reminds us, are of two kinds, crystallizable, and uncrystallizable or organized; to the former belong sugar and vinegar, to the latter the different forms of the starchy or amylaceous principle, of lignin or the woody, and of gum or the mucilaginous principle. The only crystallizable product used to any extent as food is vinegar, and this is readily digested by healthy stomachs. Our author, however, referring to certain states of disease in which this organ appears to lose the power of assimilating this principle, expresses his belief that "on the whole it is doubtful if mankind have been the gainers, except in convenience, by employing it in a form which is the furthest possible removed from organization and life," a denunciation levelled at this principle more particularly from its effects in diabetes.

Of the characters of these primary alimentary principles, of which it is unnecessary to rehearse the species or familiar properties, none is more remarkable than the capability they possess of assuming an infinite variety of forms, without undergoing any change of their essential composition, as illustrated in the mutation of sugar into vinegar or acetic acid. But this is not the only change,—they have the faculty of readily passing into each other under the influence of a *converting agency* existing in the animal organization into which they are introduced. Nay, still more, these principles are all susceptible of transmutation according to ascertained laws into totally new compounds; for example, the saccharine is readily convertible into oxalic acid or into alcohol, itself a version, if we may so speak, of the oleaginous principle. But such modifications of the primary or *staminal* principles, as Dr. Prout names them, though endlessly diversified, bear, as he justly points out, but a very limited proportion to the original radicles supplying them. They are either the product of glandular secretion, or excrementitious, or extra-vascular; or, in still more general terms, they form no part of the animals in which they are developed. They are however often attached to these, as for instance in the form of a shell to the Mollusca.

Dr. Prout, having laid down the law that these proximate principles upon which animals subsist are, with the exception of the saccharine, identical in their essential characters with the components of the tissues in the superior animals, draws the inferences that, first, such animals are spared the labour of forming their proximate principles from their elements, (a task devolving on inferior animals and on plants;)* and secondly, that an appropriate diet should contain more or less of all these principles. That the latter position is correct, is alleged to be further established by two additional facts of different character, the composition of milk, and the impossibility of supporting animal life upon a single proximate principle. It is true that milk, the only material distinctly and exclusively produced by nature for food, and which may therefore be regarded as the type of what aliment should be, contains the four staminal compounds; but the other fact, which has been repeatedly ascertained by direct experiment, may be and has been explained in different modes from that adopted by our author.

Such then are the materials from which animal organisms derive their means of existence, and perpetuate the admirable machine constituting them. The processes by which they appropriate these materials are comprised under the general title of assimilative. Assimilation is therefore a compound action, or rather series of actions, divided by our author for the purposes of description into two classes: the first embracing all organic manifestations of activity tending to and terminating in the formation of blood, in other words, digestion and sanguification; the second, the formation of tissue and the redissolution of this with its subsequent removal from the system. In both of these species of operation water, that essential constituent (whether chemically combined or mechanically united with them) of all organized bodies, plays a very prominent part. In the first place, by the variation of the proportion in which it enters into the composition of bodies, these are altered materially in their character as regards their alimentary nature and power. Thus, when it is combined in small quantity with any body, this is usually of a firm or stable character; when in a high ratio, this is distinguished by the opposite properties. Now, as the mutual interchange of these conditions, of strength of principle into weakness, and *vice versa*, constitutes one of the most frequent and important species of chemical processes taking place in organized bodies, and is therefore the subject of constant allusion, it is well to devise terms to express it concisely and significantly; this Dr. Prout has done by applying the word *reduction* to the change of a strong into a weak principle, *completion* to the converse process.

The process of change undergone by the food in the stomach is of the first kind; Dr. Prout considers that the semiliquefaction of the ingesta probably consists essentially in this combination with water, in other words, their *reduction*. Secondly, as the stomach forms identical chyle from food of various descriptions, it follows that it possesses the faculty

* "Plants," it has been said, "seem interposed between the soil and animal life, as laboratories for combining the elements of inert matter into substances capable of being assimilated in the digestion of animals."

of changing into one another the different simple alimentary principles, or, as our author designates it, a *converting* power. Both these processes, it is clear, are purely chemical. But, as Dr. Prout conceives, these are not the only powers of the stomach; he maintains that it possesses a *vitalizing* or *organizing* faculty, whereby it is enabled to fit the crude aliment for contact with living structure. The possession on the part of the stomach of this vitalizing power is not by any means demonstrated in the pages before us; and why the vivifying influence should not act in the second stage of assimilation, the most probable notion as it seems to us for various reasons, is not attempted to be shown. Of the nature of the peculiar actions by which the combination of alimentary substances with water is effected, Dr. Prout laments our ignorance; but the peculiar juice developed in the stomach during digestion must of course be the *reducing* agent. This juice is made the subject of examination by our author, and the question of the source of chlorine or rather hydrochloric acid, forming one of its most essential ingredients, discussed. He conceives it quite unnecessary to suppose this generated *de novo*, maintains that the common salt existing in the blood must be its source, and inclines, as of old, to the notion that the separation is effected by some modification of electricity. But as the acid of the preexisting muriate of soda is supposed to be thus disposed of, it remains to be enquired what becomes of the base from which it has been separated. Dr. Prout is at no loss in assigning the alkali its *rôle*: "the soda remains behind or is absorbed into the mass of blood, and a portion of it, no doubt, is requisite to preserve the weak alkaline condition essential to the fluidity of the blood. But the larger part of this soda is probably directed to the liver, and is elicited with the bile in the duodenum, when it is thus again brought into union with the acid which had been separated from the blood in the stomach."

Our author professes what may be called, in regard of admitted doctrine, a heterodox opinion on the lactic or acetic acid found in the stomach during the process of digestion. Instead of regarding this, as it is generally considered, as an ingredient in the composition of the gastric juice, second only in importance to the muriatic acid, he believes that it is rather to be looked on as the result of unnatural irritation, produced by disease, indigestible aliments, &c., than as a healthy product necessary to the digestive process. But for this extreme opinion no efficient reasons are given, and its probability seems to be materially shaken, if not altogether destroyed, by the experiments of Tiedemann and Gmelin, and still more distinctly so by the analysis of the gastric juice obtained by Dr. Beaumont from the stomach of St. Martin. That the oxalic, butyric, and carbonic acids are unhealthy products, when occurring in the stomach, we of course find no difficulty in admitting with our author.*

Dr. Prout next explains his views on the converting powers of the stomach; according to these views, under ordinary circumstances, the essential effects of their action are the conversion of the saccharine into albuminous and oleaginous, of albuminous into oleaginous, and of oleaginous into albuminous principles. The first of these changes he regards as by far the most important, for saccharine aliment cannot in its natural

* Dr. Prout enters into no enquiry upon the nature, properties, or action of the digestive element *pepsin*.

state form a constituent of any part of the living frame, yet so essential do its ingestion and conversion seem to the perpetuation of even the lowest forms of life, that they are performed even by vegetables. This power of appropriating the saccharine principle may be looked on as the lowest element in the process of renovation, and is probably the last that ceases to exist in an animal, often persisting long after the faculty of assimilating oil and albumen has been lost. Of the temporary and partial suspension of this power, occurring in and constituting one of the most important disturbed states in diabetes, we shall have occasion to speak at length hereafter. The mode in which the essential changes of alimentary principles are effected in the stomach are probably purely chemical; thus it is likely that the conversion of sugar into oil is brought about precisely by the series of changes spontaneously developed during its conversion out of the body into alcohol, which is nothing more than a weak oil. With respect to other changes, we are less capable of tracing their mechanism; the origin of the azote is not yet fully cleared up, though it would appear to be commonly derived from some external source. "The azote may, in some instances," says Dr. Prout, "be derived from the air, or *generated.* But my belief is, that, under ordinary circumstances, the azote is principally furnished by a highly azotised substance [organized urea?] secreted from the blood, either into the stomach or duodenum, or into both these localities, and that the portion of the blood thus deprived of its azote is separated from the general mass of blood by the liver, as one of the constituents of the bile; which secretion, as a whole, is remarkably deficient in azote." But it must not be supposed that the capabilities of the stomach are, under all conditions of existence, limited to the production of these changes: when animals are accidentally obliged to subsist upon a diet of wholly unusual character; when they are placed under what Dr. Prout calls "extraordinary" circumstances, "the changes out of the ordinary course are," to use his language, "altogether astonishing, and such as defy our utmost calculation. The assimilating organs appear even to decompose principles which are still considered as elementary, nay, to *form* azote or carbon." The vastness of this hypothesis is absolutely startling, for it ascribes to the animal tissues the power of producing something out of nothing—in fact, of *creating*: in the existing state of the natural sciences, it is almost needless to say that such doctrine cannot be healthfully entertained. Possibly the explanation of this is, that Dr. Prout does not consider the elementary character of these bodies established.

So much for the *converting* function; the *completing* and organizing processes to which the chyle is subjected in the lacteal system receive little novel illustration at the hands of Dr. Prout: we pass on, therefore, to his views on secondary assimilation. Under this head are comprised, according to his arrangement, first, the *formative* assimilation of the materials of the blood into the tissues and the products of the secretions; and secondly, the *destructive* assimilations (or secondary digestion) of the tissues, with their conversion into new principles designed for further use, or into mere excretions.

The general principle on which these processes are conducted is explained by Dr. Prout as follows: The elements composing the albumen of the blood, for instance, when this is destined to undergo a change of constitution, are subjected to such modified arrangement as ends either in the production of a single new principle with different sensible properties, or the elements are so combined as to form two or more principles, each of which is the complementary albuminous principle of the other; in different words, added to that other will produce albumen. This latter mode of decomposition, which is infinitely more common than the other, is of two kinds. By the first, a substance is converted into an excrementitious principle, and into one available for ulterior purposes in the economy; for example, albumen is changed into gelatine and hydrated carbon, capable of becoming carbonic acid on exposure to air in the lungs. By the second, the change is into two principles, both designed for ulterior offices, or both excrementitious. The first belongs, according to our author, to healthy action; the second, to disease. As an instance of the latter, we are given the mutation of gelatine into some modification of the saccharine principle and urea.

These laws, however, only apply to the essential elements* of organisms; of those bearing upon mineral elements *incidentally* present therein, Dr. Prout has only to state the inefficiency of existing knowledge. He agrees, however, with Berzelius in thinking that such incidental matters usually exist in their elementary condition in organized products, and not as binary compounds; and that the latter form is only assumed as a consequence of the destruction of the organized principle with which they may be associated. Hence, Dr. Prout would draw the important inference in pathology, that the appearance of an incidental binary mineral compound involves the idea of mal-assimilation or destruction of some organized tissue; and further, that as certain incidental mineral elements are always peculiar to and therefore characteristic of certain tissues (as phosphorus of the nervous, &c.), the tissue thus morbidly engaged may be predicated from the chemical composition of the incidental binary compound.

It is unnecessary for us to analyze closely Dr. Prout's statements on the formation of the gelatinous, albuminous, and fibrinous tissues, or, as he terms them, the processes of gelatification, albumification, and fibrification. When we have stated the hypothesis, that "when albumen is converted into gelatine, carbon is eliminated, which carbon (partly perhaps in a hydrated, partly in an oxygenated form) remains associated with the venous blood till its arrival in the lungs; when, by combining with the oxygen of the atmosphere, it becomes fully oxygenated, and is converted into carbonic acid gas, and in this form makes its escape from the body," we have extracted all the novelty this section affords. For the arguments supporting the view here brought forward, the author refers his readers to his Bridgewater Treatise.

Dr. Prout is rather chary of statements respecting the character of the ulterior changes belonging to the class of destructive assimilation undergone by the three essential proximate principles. He is, however,—persuaded for reasons which, as he alleges, the practical nature of the book before us prevents him from entering upon,—of opinion that one mode in which the *gelatinous* tissues become effete is by their conversion into two classes of complementary principles, of which urea, or its equivalent, is

* The term *essential* element is applied by Dr. Prout to oxygen, hydrogen, carbon, and nitrogen; on the remaining elementary substances of animal organisms (amounting to some fifteen or sixteen) he confers the name of *incidental* element.

one, and the saccharine principle, most commonly in the form of lactic acid, is the other. And, again, he believes that one of the crystallizable principles produced from effete *albumen* is lithic acid, most usually in the state of lithate of ammonia; the presumed complementary substances of which will be alluded to hereafter. On the ulterior changes of the oleaginous principle no theory is hazarded.

In the enquiry into the general pathology of the primary and secondary assimilating processes, to which the volume next introduces us, the aberrations of each of these from the healthy state are slightly sketched. An idea of the character of deduction drawn by Dr. Prout from his investigations on this branch of the subject may be had from considering the following train of hypothesis. When the appetite has been over-indulged, and more food swallowed than the wants of the system require, a deposit is commonly, as all the world knows, observed in the urine. Dr. Prout conceives that in this case the *reduction* and *conversion* of the surplus quantity of food takes place, but that the stomach fails in *vitalizing* it, and that this non-vitalized portion is permitted to pass through the kidney in the form of lithate of ammonia. But this mode of relief requires the kidneys to be healthy; now Dr. Prout supposes that the necessary state of health does not exist in certain individuals during youth, and that either from this cause, or from original weakness of the assimilating organs, the relief referred to is not obtained under the supposed circumstances of surfeit. In such subjects "the imperfectly assimilated chyle," continues our author, "either does not undergo the necessary changes in passing through the lacteal system, by which chyle is converted into blood, or is malconverted into the comparatively insoluble pseudo-albuminous matter of struma, which, in passing through the lungs, lays the foundation (perhaps at first mechanically) of tuberculous deposition and future accretion." This urinary theory of phthisis might, to a certain extent, be made a question of direct experience ; at least, it would be necessary, *in limine*, to ascertain whether the urine of scrofulous subjects, after such indulgence as would lead to lithic deposit in healthy subjects, is free from abnormal deposition of that salt. But our author does not appear to have considered the point in this light, and devotes a note to some further speculation, supported by such lame arguments as appear in the following quotation : "Strumous, lithic acid, and gouty diseases are all results of mal-assimilation of the albuminous principle, either primary or secondary, and often gradually run into each other. Thus gout and struma are frequently, if not always associated; and the gouty chalk-stones of old age may be considered as little more than modifications of the scrofulous tubercle of youth, both being alike formed from mal-assimilation of the albuminous principle." The unproved allegation, that the offspring of gouty persons are more subject than others, *cæteris paribus*, to phthisis, and the fact that tophi are often accompanied with renal disease, are the only statements, wearing the semblance of argument, adduced in support of these curious hypotheses. The notion that scrofula, gout, and phthisis are mere modifications of each other has been sustained by an equally unsatisfactory style of argument by Sebastian of Groningen.*

A distinction between the danger of secondary diseases depending upon

* Brit. and For. Med. Rev., vol. VIII., p. 242.

primary or upon secondary mal-assimilation is ingeniously pointed out and illustrated by Dr. Prout. Those originating in the primary aberration are, "in general," of much less formidable character than those of the other class. Thus the appearance of lithate of ammonia in the urine (a phenomenon often pressed into the service of illustration) is one of the most common effects of slight error in diet; but that salt is deposited in some cases when no food has been taken into the stomach;—now the latter occurs in certain fevers, and other severe constitutional diseases.

A chapter on the general characteristics of the blood, the bile, and the urine next follows. These cannot be said to contain much not already made public, either by the author himself or by others; one or two points, however, require notice. Of these is the modification of the opinion formerly advocated by the author respecting the cause of the deposition of free lithic acid in the urine. Dr. Prout now believes the free acid, or precipitating agent, to be the lactic; in some instances, it is true, the mineral [sulphuric, muriatic] or other acids may be the remote cause of the precipitation; [how so if these acids are present only in saline combination and the phosphoric alone as a *super*-salt?] but this is only from their separating the lactic acid from the base with which it is combined, and so allowing it to be the immediate organ of precipitation. Dr. Prout, however, thinks that in the greater number of cases of lithic-acid gravel, lactic acid is secreted in excess, either separately, which is comparatively rare, or combined with urea, which seems to be the rule. Here he appears to reject by implication the opinion of MM. Cap and Henry, that urea always exists with urine in combination with the acid in question.

It will be perceived, as an inference from the train of argument just stated, that Dr. Prout holds unchanged his former notions respecting the combined condition of the lithic acid in the urine. In favour of these he reiterates the evidence long familiar to the profession, without even alluding to the important microscopical observations of Donné and Quevenne on the subject; and, judging from the incomplete view given of them, appears to have become acquainted with the doctrines of Duvernoy and Wetzler rather from a recent work on urinary complaints than from the writings of those persons themselves. Dr. Prout assumes to himself merit for "avoiding all controversial points," but he means the discussion of these; so far from avoiding such points (unfortunately we know not the branch of medicine where such system of conduct could be pursued,) he settles them invariably *ex cathedrâ*, without the least apparent misgivings as to the rectitude of his decisions. However grateful it may be to some readers to meet with a conviction so strong that it almost displays itself in dogmatism, we, for our parts, feel persuaded that it diminishes the real usefulness of the volume.

Dr. Prout, like M. Rayer, has never been able to satisfy himself that casein may, under any circumstances, become a constituent of the urine. In commenting upon the statement of Liebig, that xanthic oxide differs from the lithic acid only by containing one proportion less of oxygen, the author seems inclined to doubt its accuracy, because, in the first place, he knows of no apparatus or means of operating capable, where azote is concerned, of unequivocally deciding upon the presence of one proportion of hydrogen, or even of oxygen in a complicated body; secondly, because the foreign chemists are usually in the habit of following the calculation of Berzelius, as respects the combining weight of carbon, and therefore of making it considerably higher than six, its real value, as is now recognized both at home and abroad.

It is affirmed in these pages that the existence of the prostatic secretion in the urine may be detected by certain particular appearances. We were the more anxious to learn what these particular appearances might be, because M. Rayer has recorded his inability to point out any essential characteristic of such impregnation; but Dr. Prout singularly enough makes no attempt to impart his knowledge to his readers, on the ground that "the appearance in question can hardly be so described as to be made intelligible to those who have not attended to urinary phenomena." But surely there are some persons at least who have made these a study; why is the information withheld from them?

If a general view be taken of the constituents of the bile and urine, one of the most striking points of distinction between them will be found to be the comparatively great number of oxidized and acidified principles existing in the latter. The character of the function of the kidneys, of which the acidity in question is evidently the result, is by Dr. Prout termed positive; that of the liver negative. The liver depurates the system of unassimilated and superfluous oleaginous matters, and of those portions of blood deprived of azote and of vitality; the kidneys, on the other hand, exercise the same action with respect to the albuminous principle and the mineral matters incidental to them. And it is further pointed out that the changes produced by the liver on the principles it separates, are in some measure of an organizing kind; that is, the principles separated retain some of their vitality for ulterior purposes; whereas the function of the kidneys is of a disorganizing kind, all materials passing through those glands being perfectly deprived of vitality. In certain states of disease, as we shall hereafter see, the character of these functions is in the instance of each organ changed.

We have now made the reader acquainted with the more important part of Dr. Prout's preliminary matter; and analyzed this with sufficient closeness to convey a general impression of the character of the declared laws and of the inferences upon which the doctrines broached in the body of the volume are based.

The main part of the treatise is divided into two books, devoted severally to the history of "functional," and of "mechanical" diseases. The division is, however, most imperfectly retained in the sequel, for organic lesions of the most profound character (such as those known under the title of "Bright's disease,") are considered in connexion with those of functional character. But we have no space for a discussion upon errors of classification. Under the head of functional diseases are described "diseases arising from the deranged operations and less obvious lesions of the assimilating and secreting organs." Now, as the assimilative processes bear on four alimentary principles, it follows that there are four classes of functional diseases, of which those dependent on derangement of aqueous assimilation are first examined. In commenting upon the distinctive characters of the *urina potûs* (or of assimilation, as he proposes to term it,) and the *urina sanguinis*, the author insists upon the necessity—and that a truth so obvious should be habitually disregarded in practice is a sufficient proof of the imperfect use to which we put the means of diagnosis nature places within our reach,—of examining in cases of disease a specimen of each of these varieties. Of the derangements connected by Dr. Prout with morbid states of aqueous assimilation, being simply excess or deficiency of urine, he very correctly abstains from speaking at this stage of his progress, inasmuch as these conditions are simply symptomatic of, and do not in themselves constitute, disease.

The views developed in the next section on the pathology of saccharine assimilation were some time since made known in outline to the profession.* The labours of the intervening years have, in the estimation of the author, verified and extended the applications of the doctrines then broached. Dr. Prout's argument runs thus: as the blood in health contains no sugar, and as the latter principle forms an ordinary part of human food, it follows that by the operation of the primary assimilating functions it must be converted into some of the constituent principles of the blood. And if the function of *sugar-conversion* (if the phrase be admissible) be allowed to exist, the reality of aberrations from the healthy state by derangement or suspension of this function is implied. Experience proves what reason points to,—the *suspension* of saccharine conversion is unequivocally shown to exist by the presence of sugar in the blood of diabetic subjects; its *derangement* made manifest by the occasional existence of oxalic acid in the urine of persons who had not ingested any of that substance with their food. The process in respect of diabetes is this: First, it must be remembered that the reduction of all forms of the saccharine principle appears to be accompanied with the development of a low sugar, easily convertible in the healthy stomach into albuminous and oleaginous matters. This change takes place speedily; the presence of sugar in the healthy organ is therefore with difficulty detected. In the diabetic stomach, on the contrary, where the reducing function is morbidly active, while the converting is suspended or paralysed, sugar is discoverable in abundance. "The first step in the derangements producing the disease called diabetes does not consist, as some have supposed, in the development of sugar in the stomach, which is a natural process, but in the greater or less destruction of the converting, and consequently of the still more important organizing functions of the assimilating organs." Upon this statement of Dr. Prout we would, in the first place, observe that the phrase "as some have supposed" is inapplicable to the existing state of belief. Mr. Mac Gregor's experiments clearly showed that sugar is contained in the healthy stomach after an ordinary meal of animal and vegetable food, and therefore, as it has been correctly put by Dr. Willis, that "diabetes is actually disease only because the degree of a natural process is surpassed." There are two principal questions to be put with respect to Dr. Prout's theory: is it necessary, is it involved by the established nature of things? secondly, if not, is it adequate? We can find no difficulty in answering the first query in the negative; there is no known fact demonstrating the impossibility of, or even rendering improbable, the conversion, under the supposed circumstances, of gelatinous and albu-

* Gulstonian Lectures; Medical Gazette, vol. viii. 1831.

minous principles into the saccharine. How are we justified in affirming that a modification of the *class* of principle may not be effected in a morbid state of the digesting agent, or of the system to which it belongs, when an interchange of principles of the same class is in health so easily accomplished? Surely the possible assumption of such power of conversion on the part of the stomach can, with no colour of consistency, be denied by a physiologist who, as already mentioned, believes that that organ can, under "extraordinary circumstances," take upon itself the office of a creator and form elements. The doctrine, in this point of view, evidently presupposes the existing power of fathoming the operations of vital chemistry to be infinitely more vast than stubborn facts, encountered on every side, will allow us room for a moment to dream. But, secondly, is the theory adequate; will it explain all the phenomena of the disordered chemistry under consideration? We think not; and for this reason. Mr. Mac Gregor, after having produced vomiting and purging in two individuals, one healthy and the other diabetic, fed both for three successive days on beef and water; the product of vomiting at the end of that period, and three hours after the last meal, furnished evidence, by fermentation with yeast, of the presence of sugar in the case of the diabetic subject, none in that of the healthy. Now, the theory of non-conversion cannot stand here, for no saccharine or vegetable matter was swallowed; hence we have positive proof that the stomach acquires the power, under certain conditions, of evolving sugar from organic non-saccharine combinations. While, therefore, we fully admit the fact of the non-conversion of the sugar produced in the stomach, we cannot, with Dr. Prout, suppose *all* the unnatural sugar therein contained to be the result of non-conversion of the quantity of sugar which would in health be developed from the same materials: in other words, the fault is a double one; *active*, in the power the stomach acquires of producing sugar from materials which would not supply it in health; *passive*, in the loss of its normal share of converting faculty. Besides, a point of no mean importance, which appears to be generally forgotten, is, that the appearance of the sugar in the stomach, however induced, is but a symptom, and cannot constitute the essence of the disease. Whatever be the change in the tissues of the stomach leading to it, whether microscopical or otherwise (for that such exists cannot, in our minds, be made matter of doubt), Dr. Prout seems to think it of not the remotest consequence, for no allusion is made to the point in his chapter.

The general causes of saccharine mal-assimilation are next examined. The internal or predisposing are most generally innate or inherited, according to Dr. Prout. The external exciting (in every instance subservient to the predisposing) are cold, moisture, and malaria. Although Dr. Prout is unwilling to pronounce diabetes a malarious disease, he has no hesitation in expressing his belief that almost all forms of disease connected with the development by the secondary assimilating processes of oxalic, lactic, and other abnormal acids, are more frequently excited by malarious than other external influences. His attention was first directed to this point by seeing in quick succession several well-marked cases of oxalic-acid diathesis from a malarious district. The inference drawn from this circumstance was confirmed by the occurrence of the appearances characteristic of oxalic instead of lithic acid in persons affected with indigestion or "cold" after the cholera; a disease which our author believes to have been of malarious origin from certain facts, for which we must refer to the original. Dr. Prout has sometimes imagined that the urine has not completely recovered (1838) its former condition since the occurrence of that epidemic.—A most important order of causes is diet. Under this head nothing novel, however, is communicated, except a diatribe against the use of tobacco in every form, as if not distinctly inducing the development of oxalic acid, at least that "of some analogous and equally poisonous principle." We confess we cannot consider Dr. Prout's reasoning strong here, though we are equal haters of the weed with himself; as, however, he has *known* "inveterate snuff-taking" lead to "malignant disease of the stomach and liver," there is every excuse, of course, for his warmth, although it be not warranted by mathematical evidence.

The confusion arising from the application of the word diabetes to all affections attended with considerable discharge of urine is justly animadverted on in these pages: their author proposes to restrict it to cases in which the urine is saccharine. The increased flow of urine is the circumstance which commonly directs the patient's attention to the urinary organs; and, as Dr. Prout remarks, "a saccharine condition of the urine exists in gouty and dyspeptic individuals much oftener than is supposed, and hundreds, who are quite unaware of it, pass many years of their lives with this symptom more or less constantly present." We know this to be a fact ascertained by other close observers of the urinary organs also. It follows from this that the physician must always experience much difficulty in determining the period of origin of diabetic attacks; Dr. Prout, however, conceives that, "by enquiring minutely as to the period when the urine was last observed to be turbid," he has several times traced attacks very nearly to their origin. He believes it probable that, at the time the urine becomes clear, after such turbidity, in patients labouring under diabetes, its saccharine condition may be considered to have set in, or, at least, to have become confirmed.

Dr. Prout remarks, "Diabetes very frequently (as far as my personal experience goes, *always*) accompanies carbuncles and malignant boils or abscesses allied to carbuncles;" a fact mentioned by Cheselden* and others of the older writers. These cellular inflammations, however, he is not quite prepared to consider as causes of the affection; and, if he were permitted to draw a general inference from his own observations, he would say "that diabetes usually *follows* cutaneous affections, and accompanies (perhaps *precedes*) the affections of the cellular tissue." The subject is curious, and has, at least in an allied form, engaged the recent attention of a distinguished continental surgeon.†

The symptoms of the affection are lucidly described: novelty is, of course, not to be expected. The absence of any important change in the kidneys is affirmed.

Our author enters very fully into the question of treatment, commencing with the important topic of diet. Admitting that an animal diet—as, indeed, follows from his own principles—ought to form an essential principle in the treatment, he considers a certain proportion of

* Anatomy, p. 139; fifth edit.

† M. Civiale.—See Brit. and For. Med. Rev., vol. X., p. 574.

farinaceous matter proper. This recommendation is founded upon the position already stated, that the assimilation of the saccharine principle is one of the last functions that becomes extinct in animals, and the most essential for the continuation of their existence. This may be very true, but what appears to us much more important is the experiment of Mr. Mac Gregor, proving directly, as might be supposed à priori, that the quantity of sugar produced in the stomach is much reduced under abstinence from vegetable products. However, we do not mean to contest the secondary importance of Dr. Prout's plan; the craving after farinaceous matter, such as we have ourselves witnessed it, becomes so irresistible in individuals submitted for a length of time to purely animal sustenance, that it seems flying in the face of nature to withhold it altogether. Yet, on the other hand, we may dearly pay for yielding to these importunate solicitations, however natural they may appear: long since, Rollo observed that a few mouthfuls of biscuit reinduced the worst symptoms in a subject whose condition had been materially improved by the animal diet. The hint thrown out by Dr. Willis, of mixing perfectly indigestible vegetable matter with strongly nutritive animal diet, founded on the well-known fact of the unfitness of very concentrated aliment for the sustenance of man, seems to us not to be despised. Every crystallizable variety of the saccharine principle is absolutely inadmissible: Dr. Prout has known "the use of a few saccharine pears undo in a few hours all that he had been labouring for months to accomplish."

It must be considered matter of regret that Dr. Prout has, throughout his volume, neglected to notice the remarkable announcement of Schwann and Müller, that artificial digestive fluid, though it perfectly dissolves starch, will not convert this principle into sugar unless saliva be added. Those who believe that the gastric error in diabetes consists, either wholly or in part, in abnormally forming sugar may feasibly make trial of the effect of non-insalivation of the food. The only objection to this seems the difficulty of accomplishing it, for the use of an œsophagus tube is a sad drawback; and, besides, the absence of saliva might, and probably would, entail some new description of disorder. The suggestion, which originated with and displays the practical shrewdness of Dr. Willis, nevertheless, deserves consideration.

Dr. Prout continues, by enforcing the importance of moderation in food. He states that he has found more relief follow the temperate use of and more support given by porter than by any other means whatever; he recommends fluids to be taken tepid. He has no confidence in any medicines recommended as specifics; prescribes venesection under the circumstances in which others have found it beneficial, or leeches to the epigastrium, if there be tenderness there; has seen no permanent benefit derived from the use of purgatives; regards the exhibition of diaphoretics as important, but does not lay the stress upon the use of the vapour bath, which well-ascertained facts warrant; acknowledges the value of opium as a sedative, but deprives it of the shadow of a claim to be regarded as capable of removing the saccharine condition of the urine, and points out that its continued use has the effect, in the end, of making patients confirmed opium-eaters; believes a combination of sedatives with astringents and tonics occasionally useful, and is disposed to think very favorably of phosphate of iron particularly, as the representative of one class of these medicines. In commenting on the treatment of the complications of the affection, and among these of hepatic disorders, the learned author takes occasion to add very materially to the value of his volume, by introducing some admirable strictures upon the abuse of mercury in such treatment. We agree to the fullest point in the spirit of these remarks, and only regret that their length prevents us from finding room for them in full. The following short extract will suffice to direct attention to them: "I can only say that a large proportion of the most inveterate and dyspeptic and urinary diseases which I have seen have been distinctly referrible to the use of mercury." Well may the physician, who has had experience to this effect, stigmatise those who, "to save themselves trouble, and, at the same time, to gain the doubtful reputation of being decisive and quick in their practice, resort to mercury, without due regard to its remote consequences."

When the urine of an individual is transparent and remarkably free from sediments, of a pale citron-yellow or greenish hue, and of a specific gravity oscillating about and near to 10·20, there exists a concourse of circumstances sufficient to lead the experienced observer to suspect the existence of the oxalic-acid diathesis. Unless corroborated by the presence of such gastric and general symptoms as usually attend that diathesis, it is however more likely that the characters described depend on the temporary influence of certain articles of diet, &c. And caution in forming our judgment is the more necessary, as in the statement of symptoms given by Dr. Prout, we have scarcely been able to detect a single particular which might not exist under very different conditions of the system. Of those apparently most characteristic "a tendency to periodical discharges of dark-coloured blood, both from the rectum and bladder," is the most remarkable; we mean, of course, of those occurring before the period when the presence of a calculus in the kidney has become matter, if not of certainty, yet of strongest presumption. It has long been known that hemorrhage is more frequently produced by oxalate of lime calculi than by others, an explanation being found in the roughness of its mulberry-like surface; but Dr. Prout is disposed to place it more especially in the diathesis itself. We have already noticed the belief of this writer respecting the influence of malaria on the diathesis in question; now at the time of the cholera a greater number of cases of hemorrhage from the urinary organs occurred to him than in any previous period of equal duration; at the time, these cases were taken for examples of calculous or malignant disease, but the bleeding ceased under circumstances precluding a belief in the existence of either of these forms of disease: hence the inference that it depended on the oxalic-acid diathesis.

The non-assimilation of oxalic acid taken as food (in sorrel, tomatos, rhubarb-stalks, &c.) or the real assimilations of saccharine aliments, and in extreme cases, perhaps, of the albuminous and oleaginous principles, may be regarded as the proximate causes of the disease.

The diet applicable in this diathesis is closely similar to that suited to diabetic patients. Porter in small quantity or dry sherry, even hock and claret in similar moderation, may be taken. The quality of the water used is of the last importance; it is obvious that persons labouring under this diathesis, who drink *hard* water containing lime in solution, are exceedingly liable to have an oxalate of lime calculus. By adopting a careful diet, and taking a course of mineral acid (muriatic,) three or four times a year, on each occasion for about a month; (or, more accurately speaking, until lithate of ammonia or lithic acid begin on each occasion to appear in the urine) Dr. Prout has seen the diathesis gradually subdued and at length removed altogether.

With the following section, devoted to the subject of abnormal development of lactic and allied acids in the stomach, we shall not long delay.* The development of this acid in excess, by the *primary* assimilating processes, is stated to constitute one of the most common and troublesome forms of dyspepsia; by the *secondary*, to give occasion to many serious and most painful affections, of which rheumatism and neuralgia are the most remarkable. The last portion of the chapter is so intricately hypothetical that it would require some pages to explain what, as we conceive, is by no means worth the space. The practical part of the section is good, and there is valuable truth in the considerations on the exhibition of alkalis.

The derangements of albuminous assimilation next engage Dr. Prout's attention; and as these are most prominently marked, or at least best identified by the changes they induce in the urinary secretion, he makes them the basis of his description and arrangement. These derangements are of four kinds, according as they are accompanied by (*a*) excess or deficiency of urea; (*b*) by the presence of albuminous matters in the urine; (*c*) by that of lithic acid and its compounds; (*d*) by that of cystic oxide.

Diuresis with excess of urea is, by Dr. Prout, regarded as a rare affection; for one such case he has himself seen twenty of true diabetes; nevertheless he suggests that the disease may, in some cases, pass unnoticed from the patient's not applying for advice, until it has merged into diabetes or some other formidable affection.

Dr. Prout retains his former division of albuminous urine into the chylo-serous and serous. Of the remarkable affection characterized by a discharge of milky-looking urine, he has now had an opportunity of seeing more or less of thirteen cases, but he does not appear to have ascertained any new facts on the subject since the publication of the lectures already referred to; and the important parts of these have, in various forms, been brought before the public. In seeking for the proximate cause of the affection, he is led to suppose "that the chyle from some derangement in the process of assimilation is not raised to the blood standard; and consequently, being unfit for the purposes of the economy, is, agreeably to a law of the economy, ejected through the kidneys; but these organs, instead of disorganizing or reducing it to the crystallized state as usual, (that is to say, instead of changing it into lithate of ammonia,) permit it to pass through them unchanged. That this is a just view of the matter cannot, I think, be doubted; for if the chyle was properly converted into blood, not chyle but blood ought to

* Does not Dr. Prout contradict a previous opinion in affirming here, that the presence of muriatic and *lactic* acids appears to be necessary to the accomplishment of the reducing function of the stomach; for at page xxvi. we read, "that lactic acid is rather to be considered as the result of unnatural irritation, than as a healthy product *necessary* (the italics are the author's) to the digestive process?"

be thrown off by the kidneys." Of the essential proof of the presence of chyle, namely, that of the chyle-globule, Dr. Prout says nothing. Respecting the treatment, his experience furnishes him with no suggestion of value; counter-irritation in the form of seton may do good at first, but the complaint returns after the system has become familiarized to the stimulus of this; the same may be said of mineral acids and of opium. It is consolatory to know that the general symptoms are frequently of trifling severity, that such indication of the existence of the affection may be altogether absent, and that temporary spontaneous disappearance of the complaint is far from uncommon. The narrative of the case of a woman, commenced in the former edition of the work, is brought to a conclusion in the present; she died in an emaciated state in 1836, after having laboured under the affection for nearly twenty years.

Pass we now, under the guidance of Dr. Prout, once more to the subject of albuminuria, a morbid state so fully discussed of late in the pages of this Journal that our task will be now limited to an exposition of the original matter, speculative or otherwise, of the author. Diseases of this kind are by him considered "as of two principal kinds or species only; one of which may in general terms be said to be of an *acute*, the other of a *chronic* character." The former includes two varieties, the latter eight; all ten pass into each other by imperceptible degrees; the only distinguishing feature in the urine being that, in the two acute, that fluid lets fall lithate of ammonia on cooling, while in four of the chronic species this phenomenon never occurs. Before entering upon the particular history of these varieties, Dr. Prout delivers his opinion upon an important question of general pathology. It is a mistake, he conceives, to consider, as French authors more particularly do, inflammation as almost the only cause of disorganization. That inflammation is the immediate cause of death in most of the diseases of the kidney distinguished by the suffix *itis*, he does not deny; but that even when qualified by the epithet chronic, the word rightly designates that comparatively quiescent state of the kidneys which immediately preceded the fatal inflammatory attack, he refuses to believe. All organic affections may, he maintains, "be supposed to arise from two distinct causes, degeneration and inflammation." By the former is meant that slow and gradual change inseparable from advancing age, and therefore normal; though it may be induced abnormally by inherited and innate, or by acquired weakness. The causes of the latter may be slowly acting, such as continued errors of diet, or acute, such as inflammation, severe accidents, &c. From this view Dr. Prout draws five inferences; four of these are so clearly involved by the premises, that there is no necessity for stating them. The fifth proclaims that "the appearances presented after death under these circumstances are quite useless in a pathological point of view, because it is impossible to distinguish what is due to degeneration and what to inflammation." Finally, Dr. Prout has not seen this doctrine, though it may so exist, exposed *todidem verbis;* he, however, confesses his reading on such subjects has been limited, as in consequence of the neglect of these and other important distinctions by authors, "their works present a confused and unphilosophical jumble." From this flattering compliment to the existing tribe of pathologists and pathological anatomists, let us turn to the theory, the conception of which has demonstrated to Dr. Prout the worthlessness of all existing pathological information. It may be stated, first, that to the term degeneration there is this strong objection, that it has been, and still is, used by numerous writers in a completely different sense. Next we would ask, does it not bespeak an unwarrantable degree of self-confidence in an author to state a doctrine presumedly subversive of pre-existing knowledge, without attempting to support it by an elaborate series of arguments, and above all of facts? The question is simply this: do the characters of what the experience of all countries has united in terming chronic inflammation, exist in certain cases? Secondly, if so, are these characters phenomena which arise, and only arise, from a previous condition of tissue, known conventionally as inflammation? Dr. Prout avers the negative of both positions; but his intimate conviction of this seems to be the proofs with which we must satisfy ourselves of its correctness. It would be less unfortunate that much of Dr. Prout's chapter turns upon this mode of classing organic changes, did he tell us what this degeneration is, what the characters by which it may be recognized: but nothing of the kind is distinctly done. The subject of serous urine is, in pursuance of these views, presented by Dr. Prout in the following manner:

| | |
|---|---|
| Species *a*, serous urine; the kidney in a state of health. | Var. 1. Quiescent.<br>Var. 2. Inflamed. |
| Species *b*, serous urine; the kidney in a state of degeneration. | Var. 1. Quiescent.<br>Var. 2. Inflamed. |

We shall, with as much brevity as possible, state the leading characters assigned to these varieties.

Spec. *a*. var. 1. Here we have the healthy kidney quiescent; is albumen ever present in the urine under such condition of these organs? Our author admits that it is, for example, as a consequence of admixture with blood, or with that species of pseudo-serum thrown out during inflammation of the mucous surfaces. Again, a suspension of the healthy disorganizing function of the kidney (that whereby it converts albumen into lithate of ammonia) may take place when some condition of the kidney approaching to the inflammatory, coexists with certain derangements of the system at large. Such is supposed to be the state of things when albumen appears in the urine in consequence of eating cheese, of using cantharides, &c. Further, as albuminuria is not always produced in all subjects by such causes, he infers that "in the person liable to be so affected, there exists a sort of latent predisposition (incipient degeneration) to kidney disorder." Functional derangements, productive of albuminuria, are thus admitted; but it is advanced as "not improbable, that such functional derangements may partake of the character of incipient disease." It will be perceived, that so far as Dr. Prout states the result of observation, his statements agree with the views taken in the articles on Renal Pathology in this Journal; that he should differ from us, when he wanders into the region of speculation, we can really feel no concern.

Spec. *a*. var. 2. Here we have the healthy (that is, the non-degenerated) kidney seized with inflammation: the urine is albuminous, of deep colour, below the natural standard in quantity, varies in specific gravity from 1018 to 1030, or more; sometimes contains blood and deposits a deep brownish-red sediment of lithate of ammonia. This state of the urine is accompanied with a tendency to a species of anasarcous œdema, known as inflammatory dropsy. We need go no further to show that Dr. Prout here has in view the first stage of the *néphrite albumineuse* of Rayer, in other words of Bright's disease; but as there is no formal description of the anatomical characters of the disease, how are we to understand that Dr. Prout has personally ascertained the reality of an inflammatory condition; a point hitherto distinctly contended for by Rayer alone? The proximate cause of the affection our author places in an inflammatory state of the system generally, but involving the kidneys in particular; an hypothesis conceived in order to explain the difference in the train of symptoms induced by this disease and by simple acute nephritis. Persons who do not die during the acute stage of the disease "generally perish sooner or later with all the symptoms of degeneration of the kidneys and serous urine, in their worst forms:" the affection is thus further identified with the first stage of Bright's disease. "Allied to it," says Dr. Prout, "is the anasarca following the exanthemata." Bleeding, the exhibition of diaphoretics, and after awhile of mild diuretics, constitute the treatment recommended. When the disease has originated after scarlet fever, it has occasionally, as we are reminded, been treated with large doses of calomel, "which no doubt contributed its share towards the production of the chronic forms of the disease under which the patient laboured."

In species *b*, the kidney is degenerated. Upon the character of the degeneration particular ages appear, it is alleged, to exercise the most fundamental and important influence. When renal disease occurs before the age of forty, it is almost always, we are told, to be considered as acquired *de novo*, or as resulting from inherited predisposition or struma; when after that age, partly from long-continued and slowly-acting causes giving occasion to gout, &c., and partly from natural decay of the vital powers. These facts, as they are called, supply the inference that *anæmotrophy* will constitute the leading feature of the diseases of the kidney in early life and *hæmotrophy*, "from the plethora produced by the daily use of a generous and stimulating diet, in advanced years."* It is not however contended that hæmotrophy does not exist in early life and anæmotrophy in old age; but these conditions are then coupled with disorganization. These two states of defective nourishment are, in Dr. Prout's view, so practically important, (little does it bespeak of perfection in our science and our art, when that can be pronounced practically important which is essentially hypothetical,) that he makes it the basis of his arrangement of renal affections connected with serous urine. Let the reader bear in mind that Dr. Prout does not by any means appear to have, or affirm that he has, observed, with such frequency as to exclude the idea of accidental coincidence, the fact of these anatomical conditions being more or less peculiar to a given age; but that imagining from such *à priori* considerations as are stated above that such *should* be the case, he finds no difficulty in endeavour-

* Anæmotrophy and hæmotrophy mean respectively excess and deficiency of sanguineous nourishment, and therefore differ both from anæmia and hyperæmia, and from atrophy and hypertrophy.

ing to persuade his readers that it is so. Here is his arrangement based on this bipartite division of renal nourishment:

| | | | |
|---|---|---|---|
| Species *b*. Kidney degenerated. | Sect. I. In a state of Anæmotrophy. | Subspecies *a*. The kidney in a state of *organic change*, but without any *visible* derangement of its ultimate structure. | Var. 1. Quiescent.<br>Var. 2. Inflamed. |
| | | Subspecies *β*. The kidney in a state of disorganization, i.e., having its ultimate structure more or less visibly destroyed. | Var. 3. Quiescent.<br>Var. 4. Inflamed. |
| | Sect. II. In a state of Hæmotrophy. | Subspecies *γ*. The kidney in a state of *organic change*, &c., as in *a*. | Var. 5. Quiescent.<br>Var. 6. Inflamed. |
| | | Subspecies *δ*. The kidney in a state of disorganization, &c., as in *β*. | Var. 7. Quiescent.<br>Var. 8. Inflamed. |

Considerable confusion arises from Dr. Prout's manner of examining the phenomena of these different forms of disease; we must, however, follow the order he has adopted, in such observations as we feel called upon to make. He first considers the state of the urine, then that of the kidneys, (even this very order of priority exhibits the author's inclination to elevate the importance of symptoms at the expense of lesions, and thus, *pro tanto*, make causes subservient to their effects,) together with the causes of the two quiescent varieties 1 and 3. He then follows the same plan with regard to the other quiescent varieties 5 and 7; and, lastly, considers the diagnosis, prognosis, and treatment of the four collectively. A different order altogether is observed in examining the inflamed varieties 2, 4, 6, and 8.

Spec. *b*. Sect. I. The kidney is anæmotrophous and quiescent. The diseases comprehended in this section may exist under a variety of forms, but for all practical purposes may be reduced to the two subspecies *a* and *β*. In the former the urine is not habitually, but from slight causes becomes more or less serous, proving "that the kidneys *must* be in some incipient condition of organic change, though their ultimate structure be not visibly deranged." If advanced by a pathologist of the organic school, the clause quoted would not involve a *petitio principii*, provided the fundamental dogma of that school be supposed to be granted; but Dr. Prout, in thus expressing himself, begs the question at issue most completely; and besides, shows how easily we may persuade ourselves of the reality of the doubtful, when we suffer theory instead of observation to be our guide. In truth, what Dr. Prout had but a few pages before hesitatingly proposed as "not improbable," is now boldly announced as a necessary truth.

Subspec. *a*. Var. 1. Our author's experience "assisted by analogy," leads to the following very unsatisfactory exposition of the state of the kidneys in this variety. Their size is equal to, greater or less than that of the healthy organs; they are "usually" paler than natural; "the ultimate structure is not sensibly changed, yet often presents an appearance indefinably unnatural; the texture is sometimes irregularly firm or *lumpy*, more rarely softer than natural . . . . . and generally the kidney may be said to partake of that want of natural appearance and development which can hardly be verbally expressed." We confess this mode of description appears to us most extraordinary. It would, in truth, be impossible to draw from it a single definite idea as to the anatomical condition in view. How any object can appear unnatural, and yet not be sensibly changed from the natural state, we are at a loss to understand; and, above all, the idea of drawing up anatomical descriptions upon "analogy" (analogy of what?) is a novelty for which we were not prepared.

Subspec. β. Var. 3. In illustration of the state of the organ here, the author "limits his attention" to the "granulated kidney" of Dr. Bright, believing this condition the only one susceptible of description in the present state of knowledge. Hence it is to be inferred that there are other changes besides those made known by Dr. Bright referrible to the present head; but what these are, Dr. Prout himself is unable to inform us.

The constitutional symptoms of both these varieties are carefully considered by Dr. Prout; with those attending Var. 3, he joins the morbid states described as "secondary disorders" by Dr. Christison, from whose recent volume the greater part of the details on these appears to be derived. Hereto is appended an announcement by our author of his acquiescence in the notion already advanced by the Scotch writer in nearly similar terms, that individuals labouring under "granular degeneration" are "peculiarly liable to the invasion of some epidemic diseases." Not the semblance of a proof is adduced in support of this most in[illegible]rtant doctrine. Neither the necessity for, nor the difficulty of obtaining proof appear to have flashed across our author's mind. He has not reflected that the following steps would require to be taken in order to establish the assumed position: 1. To learn accurately what proportion of persons out of a given number affected with "granular" disease were attacked with a prevalent epidemic. 2. To ascertain what number of individuals out of a similar amount of population, unaffected with such renal disease, suffered from the epidemic. 3. To have evidence that both series of individuals, thus compared, were subjected to the same, or as nearly as possible the same, physical and social conditions. 4. Admitting that there were an excess of victims to epidemic disease among the sufferers from the renal complaint, to discover, by learning the amount and extent of organic disease of other kinds among the subjects belonging to the category free from "granular degeneration," whether the observed excess might or might not be attributed to the influence of the affection of the kidney simply as *organic*, and not specially as *renal*, disease. 5. Admitting that the result were in this instance also favorable to Dr. Prout's notion, it would be necessary for the substantiation of this, that the excess on the part of persons with the renal disease should be far from inconsiderable. 6. And, further, the point could not be considered to be demonstrated by the existence of even a notable excess of the kind during a *single* epidemic. Several successive epidemics should be observed with similar results, as these might in the first instance be the effect of hazard. All this may be troublesome enough; few are wont to prefer the enduring toil such mode of examining a subject requires to the comfortable beatitude of an easy chair, a well-nibbed pen, and a little of the imaginative faculty; of him, thus triply armed, conclusions are ever ready at the beck.

Spec. *b*. Here the kidney is hæmotrophous, and may be invisibly (subsp. γ) or visibly (subsp. δ) deranged. The quiescent varieties are therefore two, 5 and 7. In var. 5 the urine is characterized by being transparent when passed, becoming turbid after a while from copious separation of lithate of ammonia, and again clear occasionally from subsidence of that salt; such subsidence is, however, rarely complete; but the fluid may be rendered perfectly transparent by a very gentle heat, and frequently again made turbid from the deposition of albuminous matter, by raising the heat to 130° or 140°. The kidney is normal or not so in point of size, of deeper colour than natural, and "generally the kidneys seem to partake of that unnatural character, and forced or excessive development, *which hardly admits of description*, but which may *readily be supposed* [!] to be produced by long connued full diet, and the free use of vinous liquors upon the system in general, and upon the congested abdominal viscera in particular."

Var. 7. The disease is now established. The urine is almost always albuminous, "particularly after meals." In the more advanced stages it becomes impossible to lay down rules respecting the other properties; such as specific gravity of the fluid, as the original affection is liable to become complicated with other diseases of the kidney which modify them materially. The presence of these complications is asserted to be no bad diagnostic mark of the peculiar condition of the kidney now under consideration, as more immediately contrasted with the granulated form of disease, "in which," according to Dr. Prout's observation, as well as that of others, "actual calculous depositions, and even abscesses, may be said to be rare." The size of the kidney increases with the advance of the malady; its colour is dark, as if from vascularity; and the texture of the organ is softened. The section of the cortical substance is almost always broader than in health, and may present a distinct granular appearance, "though this is often entirely wanting, and the whole mass assumes a uniform homogeneous appearance, like that of the brain, or occasionally like that of degenerated fatty liver." Obliteration of the natural structure follows, and the cortical and tubular substances may eventually be so completely absorbed, that the once enlarged organ is reduced to a small flabby irregular capsule. Such is Dr. Prout's account of the quiescent hæmotrophous disorganized kidney. However familiar we and our readers may be with the individual appearances thus loosely described, we feel persuaded the concatenation of these must be new to them as it is to us. That an enlarged congested kidney should, in the natural order of things, become infiltrated with encephaloid matter (for this is the only lesion with which we are acquainted whereunto the phrase "possessing an appearance like that of the brain" is at all applicable) is in itself a startling affirmation; but the implied notion that it is a matter of indifference whether the original congestive enlargement pass into a state of such infiltration, or into a condition having the outward aspect of fatty liver, we should have thought the *ne plus ultra* of "analogical" morbid anatomy, were we not next told that "in the last stages" of the disease, the whole organ (previously changed into, it may be, encephaloid matter) is sometimes converted into a small cyst or capsule! With all our strong respect for Dr. Prout, we cannot consent to hold as feasible, notions flying in the face of all accepted doctrine, and can only express our very sincere regret that so truly gifted a man should have fallen into the sad fallacy of supposing that closet speculation, however profound, however varied, and however sustained, may supply the place of that essential knowledge furnished by the scalpel alone.

The discussion upon diagnosis is prefaced by some remarks upon the state of the blood in these affections; these are, however, principally a summary of the more important of the facts elicited by the researches of Dr. Christison. The diagnostic discussion itself bears upon the distinctive symptoms of the anæmotrophy of early life, and the hæmotrophy of middle and advanced age: the recapitulation of these exhibits a very striking amount of difference between the effects of the two species of lesion, as Dr. Prout has conceived them.

The prognosis of the four quiescent varieties is considered at some length. The blood, the urine, and the concomitant affections are examined as indicating the character of this. In respect of the former, we are told that when the position and relative proportions of the ingredients of that fluid are not materially altered, or altered in so far only as may depend upon an inflammatory condition, an early and controllable state of disease is indicated. But it is naturally added, that deficiency of hæmatosin, unless this can be referred to previous depletion—the influence of which on the colouring matter has been very clearly shown by Dr. Christison—as well as general diminution of the solid constituents of the blood, announce an advanced and generally irremediable state of disease (anæmotrophy.) As respects the urine, high specific gravity, deep colour, moderate quantity, *abundant* albuminous impregnation, (here Dr. Prout agrees with Dr. Christison in opposition to certain pathologists, who maintain that the quantity of albumen increases with the advance of the disease,)* and the presence of lithate of ammonia denote an incipient stage of disease: the opposite conditions, a form of anæmotrophy, from which recovery is impossible. It must be remembered, however, that Dr. Christison's experience is not to the same effect exactly in respect of the albumen, for he has observed the quantity very great in the worst stages of the disease, when fresh reaction may have occurred.

In examining the question of the compatibility of renal disease with life, Dr. Prout points out the important fact, that this depends mainly on our success in averting acute intercurrent inflammation of the affected organs; that hence upon the habits of life of the subject, upon the care with which he avoids exposure to cold and intemperance, mental anxiety, and severe bodily exertion, must our prognosis be mainly built. "I am decidedly of opinion," says our author, "that a very large proportion of those who labour under renal disease may live for many years and enjoy tolerable health; indeed better health and more comfort than are compatible with many other chronic and incurable diseases."

The remarks on treatment display Dr. Prout's practical skill in the management of these diseases in a very favorable light. Nevertheless, if satisfactory in this respect, their perusal is far from encouraging, for they show very distinctly that the highest degree of such skill coupled with great opportunity for its exercise, is unlikely, in the present state at least of *scientific* acquirement on the subject, to lead to any marked

* See Brit. and For. Med. Rev., vol. X. p. 312. Oct. 1840.

power of controlling, never of curing, these maladies. The three great points to be constantly borne in mind in the conduct of these cases, are attention to diet and regimen, the guarding against the influence of cold and moisture, and the avoiding all violent remedies. The diet should be light and nutritious, and free from stimulating matters; flannel should be constantly worn next the skin throughout the year, and, during the winter and spring, the addition of leather is advisable. Moderate exercise, always short of fatigue, and if circumstances permit, easy travelling by land or sea are beneficial. Copaiba, mercury, cubebs, turpentine, and similar medicines are to be avoided as destructive. Leeches or cupping-glasses to the loins may be indicated in the outset, and mild but efficient purgatives, if the hepatic system be congested. The gastric symptoms, acidity, flatulence, and nausea, are best relieved by prussic acid, sometimes united with small doses of the fixed alkalis, cautiously administered. The light bitters and tonics, such as the *infus. humuli*, or *cinchon.*, prepared with *cold distilled water*, are occasionally useful. If there be irritability of the bladder, the *uva ursi*, *hyoscyamus*, or the *diosma* and *pareira brava*, either alone or conjoined with acids, as the properties of the urine may indicate, are to be recommended.

Of the disease affecting individuals of advanced years the treatment is somewhat different. If there be an acute attack attended with hepatic symptoms, venesection, the judicious use of mercurials, and of the more active diuretics are advisable. Active mercurial treatment is however to be carefully eschewed, but to mild alterative doses of this remedy, either alone, or, if gouty irritation exists in the system, conjoined with the acetous extract of colchicum, Dr. Prout admits there can be no objection. When the active symptoms have abated, the diuretic effect of the remedies may be occasionally assisted by the use of effervescing waters; and if the patient have been accustomed to wine or spirits, a little sound sherry or hock, or a little hollands may be added. The urine gradually regains its natural appearance and transparency under this mode of treatment, and if then examined, will be found in many cases [?] to have lost its albuminous character; whence Dr. Prout infers that formidable renal disease has not existed. Very similar conduct is recommended in the advanced and confirmed stages of the disease. "The steady use of some diaphoretics" is declared to be now often beneficial, "provided their action be at the same time directed to the kidneys, or at least be not calculated to unduly suppress the action of those organs. One of the best I know is the citrate of ammonia, which may be variously associated, either with ipecacuanha and the syrup of poppies, or with Dover's powder, the extract of sarsaparilla, &c." An issue or a seton in the renal region is recommended for its palliative effect. We cannot, we think, close our notice of this subject better than by laying before the reader the following emphatic injunction of a practitioner whose experience entitles all his practical observations to serious consideration: "it should be constantly kept in view, that in all forms and stages of renal affections, and particularly in the advanced stages, *it is much easier to do mischief than to do good;* and that the great principles to be kept in view are to prevent evil as much as possible on the one hand, and on the other to meet promptly symptoms as they arise, particularly when of an inflammatory character."

We find little calling for particular notice in the few pages devoted to the four inflamed varieties, 2, 4, 6, and 8. The writings of Dr. Bright, and more especially the Atlas of M. Rayer, are referred to as containing delineations of everything that has been observed. The reference seems to us to come rather singularly from our author; but the reason of the recommendation is that, "as by far the greater proportion of kidney diseases prove immediately fatal by the supervention of inflammatory action on their degenerated structure, the appearances, described by authors as occurring after death, present in general faithful pictures of the phenomena now under consideration." Why, we had thought that they jumbled together the effects of "degeneration" and of inflammation. But be this as it may, we wish it to be understood in respect of Dr. Prout's hæmotrophy and anæmotrophy, their relation to age, &c., that we do not take upon ourselves to deny them all foundation in truth and observation; the subject demands special enquiry. Still we unhesitatingly affirm that he has utterly failed to make out even the semblance of a case.

We need only notice some isolated points in the chapter on lithic acid deposition. The different forms in which this occurs are stated to be remarkably under the influence of age: in the earlier periods of life the tendency is to the deposition of amorphous sediments and crystallized gravel; in middle age the amorphous concrete mass commonly occurs, giving occasion to the formation of renal and vesical calculi; and lastly, in more advanced age there is occasionally a disposition to the development of numerous small lithic calculi (pisiform concretions) around minute nuclei. Although exceptions to these general laws occur, yet they are of sufficient constancy, according to Dr. Prout, to allow of the age and constitutional symptoms of a patient being predicated from a simple knowledge of the characters of the lithic matter passed by him.

The causes of lithic sediments are discussed at much length. Among the predisposing, Dr. Prout ranks hereditary influence, citing as proof of the reality of such influence, that "he has repeatedly seen the children and grandchildren of individuals who had suffered from lithic acid concretions liable to the same depositions." This affords a presumption in favour of the alleged fact, but does not prove it. Lithic deposition is perhaps so common a urinary derangement, that its occasional occurrence in successive generations is likely to be observed in the natural course of things, as a mere matter of accidental coincidence. To *demonstrate* the reality of the etiological influence in question is a more serious matter than Dr. Prout seems to imagine; but the present is not the place to discuss the subject. The existing causes are classed under the three heads of diet, exercise and atmospheric influences. Of exercise it is remarked, that if taken so as to interfere with the processes of assimilation, as for instance immediately after a meal, it is very prone to induce lithic deposition. In like manner, certain exercises affecting "the dorsal system," hard riding for instance, have been observed to produce urinary sediments in those unaccustomed to such motion. On the other hand, the want of exercise after a certain stage of the digestive process is completed is usually followed by similar consequences; indeed, of the influence of full living and bodily inaction none can entertain a doubt. A theory of the *proximate* causes of the affection is next traced by Dr.

Prout in the following manner: 1. Lithic acid and its salts are, as he presumes, and as has already been stated in this article, principally derived from the albuminous principles of the chyle, the blood, and the albuminous textures of the body; as urea and lactic acid are conceived to be produced from the gelatinous tissues. Now when on account of imperfect assimilation the chyle is not raised to the blood standard, the kidney, as Dr. Prout supposes, possesses the power of selecting and disorganizing such imperfect matters, and of converting them into the lithate of ammonia; hence the *yellow* amorphous sediments occurring from slight errors in diet. 2. During feverish affections, especially when the liver is involved, the lithate is supposed to be derived from the two sources above mentioned, and also from the deranged *secondary* assimilation of the albuminous tissues. The salt now appears more especially under the forms of the *red* and pink amorphous sediments, and is distinguished by the large quantity of colouring matter formed with it. 3. The massive forms of lithic acid originate from the same sources as the above, but when thus deposited the acid is secreted either in connexion with other acids, which set it free by combining with its ammonia, or in connexion with other bases, as soda, which compounds are of very inferior solubility to the lithate of ammonia. With regard to the general pathological relations of lithic acid and its compounds, Dr. Prout hazards the hypothesis, "that the lactic and lithic acids, considered with reference to rheumatism and gout, may be regarded somewhat in the light of *materies morborum.*" Having developed this notion within certain limits, he adds that "he does not consider it worth while to illustrate these views further, much less to enter into a formal defence of them." He can scarcely expect others to examine with any care doctrines which even the partiality of authorship cannot persuade him to be worthy of defence; the only puzzle to us is, that such imperfectly elaborated notions should be considered deserving of publication.

The great point in the prevention of the yellow amorphous sediment is attention to diet; and here Dr. Prout regards *quantity* as of much more importance than *quality*. That "any stomach may digest a *little* of *anything*, but no stomach a *great* deal of anything," is a maxim to which he attaches the extremest value. However, it need scarcely be told that even a minute quantity of articles of food, confessedly indigestible, should be carefully avoided. When the yellow sediments are very pale coloured and liable to follow slight atmospheric changes and errors of diet, they commonly denote a feverish state of the system, resembling that which accompanies phosphatic deposition. The treatment then merges into that of this more dangerous variety of urinary affection. The more valuable part of the section on the treatment of pulverulent or massive lithic-acid deposits is contained in the following summary. Dr. Prout seems to settle the disputed point of the comparative appropriateness of animal and vegetable diet, by giving the palm to neither. Certain specimens of either class, such as salted and dried meats on the one hand, and potatoes and subacid fruits* on the other, will, in some subjects,

* How does Dr. Prout account for the observed fact, that subjects tortured with gout and lithic deposition frequently experience notable relief, during the season, from free indulgence in such fruits? Here is evidently not a case of *habitual* use of the presumed deleterious agent.

produce abundant lithic deposition; while ordinary food of both classes, if taken in moderation, is innocuous in this respect. The distinction of animal and vegetable origin is probably quite out of the question in the instances of disagreement above referred to. Similar notions are expressed on the subject of varieties of wine. Persons accustomed to the "strong, brandied wines," usually consumed in this country, are almost sure to suffer from a gravelly attack, if they indulge in weak ascescent wines, such as the inferior rhenish and champagne; yet individuals, accustomed all their lives to such beverage,* or to the free use of cider or perry, rarely suffer from gravel. As regards ordinary diluents, Dr. Prout conceives weak tea to be the best, reprobates the use of malt liquor of every kind, and milk, and dwells upon the importance of using soft water, and of taking a moderate share of exercise, on foot especially, if the patient have the use of his limbs. On the exhibition of alkalies, as preventives of deposition, as solvents of concretions already formed, and as constitutional alteratives, Dr. Prout severally speaks. They cannot prevent the development of, but only remove already existing acidity; are therefore best prescribed about four hours after eating, (the experiments of Schwann seem to us to throw some doubt upon their being admissible at this period,) generally in the form of from 10 to 20 grs. of carbonate of potass; and not, as is usual, in company with tonics, though the separate use of both these classes of medicine may be highly beneficial: thus the author gives alkalies after, and acids as tonics before and between meals, "with the best effects." On the question of their solvent power, Dr. Prout "has no doubt that in some constitutions large doses of alkalies can be taken for a great length of time with apparent impunity, and so as not only to affect the urine, but even to act on lithic acid calculi lodged in the kidney and bladder;" yet he cannot regard the practice in general as a safe one, and that its application is in many instances impossible, is well known. When thus used, they are best taken dissolved in a large quantity of water, as indicated by the condition in which they exist in the Vichy and other mineral waters of that class. Of the *intolerance* of alkalies, sometimes witnessed, Dr. Prout refers to a striking example; in this case the excitement produced by their ingestion bordered on delirium or mania, and though everything strongly indicated their use, they were in vain tried in every form, and if persisted in would probably have poisoned the patient. Dr. Prout does not comprehend the meaning of the charge urged against alkalies by Huxham, Dr. Copland, Magendie, and a host of other experimentalists, that they "attenuate the blood," and therefore cannot speak on the subject. The age of the patient, inasmuch as it modifies the character of the lithic deposition, the general condition of the urinary passages, and of the tone of the digestive organs, &c., exercises a modifying influence on the treatment; this part of the subject the reader will find very usefully illustrated in Dr. Prout's volume.

The notice of cystic oxide deposition is as brief as the extent of knowledge respecting it is limited. The peculiar composition of this substance,

* We have heard it stated by medical men on the spot, that calculous complaints are unknown on the banks of the Moselle, while we have not heard such immunity claimed for those of the Rhine. The inferior Moselle wines, such as are consumed by the peasantry and boatmen, are of the most harsh and pungent acidity.

and particularly the sulphur entering into its constitution, are the grounds for classing it with principles of albuminous origin. These conditions "appear to show that its formation results either from an imperfect assimilation of the albuminous principle, or most probably from the future [subsequent] action of the kidney on such imperfectly developed albuminous principle." Among remedies, alkalies may be sometimes proper, but acids are much more frequently indicated. Of the latter, Dr. Prout has generally preferred the nitro-muriatic, which he was first induced to employ from observing that it removed the peculiar odour, apparently closely allied to that produced by the cystic oxide, occasionally connected with the urine. This acid will remove the foreign principle, while its administration is persevered in, but when given up, the urine returns to its former state. The prognosis is generally unfavorable. With the notice of this substance closes the chapter on the pathology of albuminous assimilation.

In the third chapter are discussed the morbid conditions of oleaginous assimilation. The affections thus arising, in Dr. Prout's arrangement, are first those dependent on excess or deficiency of the oleaginous principle (obesity, leanness); secondly, those originating in change in the qualities of the principle, as an illustration of which the formation of biliary concretions is adduced. The different conditions which are presumed to have an influence on the development of fat, or the contrary, such as inherited tendency, climate and locality, diet and exercise, are each considered. The mode of fattening geese, by securing them immoveably in a high temperature and cramming them immoderately with food, is cited in exemplification of the effects of high feeding, elevated temperature, and rest. Dr. Prout, in justly stigmatising the barbarous cruelty committed in the production of that epicurean morsel, the *pâté de foie gras*, observes, and there is satisfaction, we had almost said, in hoping that such is the fact, "it is probable that in this and many similar instances, the poor animals sometimes *have their revenge*. Indolent and dyspeptic individuals, who partake of these diseased and poisonous productions, can scarcely be supposed, in all instances, to assimilate them; and consequently run considerable risk in inoculating and converting their own livers or other organs into a similar mass of disease." Among the causes of leanness, Dr. Prout omits to notice shortness of the intestinal canal which, in at least one well-authenticated instance of such state, combined with monstrous bulimia, appeared to afford a plausible explanation of the phenomena. After all, idiosyncrasy seems to have as great an influence on the matter as anything else; there are individuals who would, as is their own expression, grow fat on bread and water. Respecting the functions of fat in the animal frame, Dr. Prout remarks that though, as is commonly admitted, it partakes less of the character of a living organized substance than the other fundamental principles, yet that it, or some nearly allied principle, admits of the highest degree of organization of which matter is perhaps capable, appears from the large proportion in which it enters into the composition of the cerebral and nervous tissues, in association with phosphorus and other incidental mineral matters.

Matter of more attractive character than anything contained in the section on biliary calculi induces us to pass over this subject with the statement, which throws out a hint for future enquirers, that while Dr. Prout has noticed cholesteric concretions to be most usually associated with lithic acid in the urine, (the common belief is that the two things rarely coexist), he fancies he has found the other kinds of concretion, and especially the inspissated-bile calculus originate in cases where the tendency to the formation of oxalic acid and to "malignant disease," more especially of the liver, prevails.

In his fourth chapter, Dr. Prout enquires into the pathology of the incidental matters of organized products; the latter are of two kinds, soluble and insoluble, hence the formation of two subspecies. The affections connected with the insoluble compounds, the triple phosphate, and the phosphate of lime, (the latter of exceeding rarity), are first considered. The phenomena attending the separate deposition of these salts are passed in review, but no actually novel facts are brought forward. Of the proximate cause of the deposition of the triple phosphate, our author conceives that we know but little. Yet he is persuaded from all its pathological relations, that it is connected with the inordinate consumption or malassimilation of some tissue, "intimately connected with the organic system of nerves." He asserts that when the triple phosphate is deposited in large quantity, the material of the tissue referred to (probably in a modified form) is often likewise present in the urine. This material, he conceives, is usually confounded with mucus, though perfectly distinct from that principle, and assures us that he has been long familiar with its characters; but what these characters are, he makes no effort to teach his readers. The exact nature of the tissue, during the malassimilation or destruction of which the phosphate of lime is eliminated, Dr. Prout does not pretend to define; but has no hesitation in saying, that it is quite different from that engaged in the production of the triple phosphate, and seems to have more relation to the dermoid variety of the gelatinous tissues.

A much more common derangement of the urinary secretion than the separate excess of either of these salts is the combined overplus of both. Deposition of the mixed phosphates is not often, according to Dr. Prout, an idiopathic disease, but usually depends on local affections of the urinary organs. Of this we are perfectly persuaded; but we feel surprised that our author does not distinctly mention disease of the kidney itself as among the causes of such derangement. We conceive a sufficiently strong case has been made out by M. Rayer, in favour of the opinion that phosphatic urine almost always depends, in cases where it has been the habit to ascribe its occurrence to a particular diathesis, upon chronic nephritis, to merit the careful consideration of the practitioner in the position of Dr. Prout. Nor do we think our author happy in eschewing all recognition of such inflammation as an intermediate condition between "injury of the back," and the appearance of phosphates in the urine,—in all probability the real and essential cause of such appearance.

Urine, containing the soluble incidental matters, soda, potass, and ammonia in excess, is next described. It is alkalescent when passed, of ammoniacal and peculiar odour, pale coloured or reddish, opaque from the presence of pus or mucus, usually contains a less proportion of phosphates than the healthy fluid, and as the alkaline bases are combined with carbonic acid, effervesces strongly on the addition of an acid, is deficient in urea, contains a large proportion of lithate of ammonia and soda, varies in quantity and in specific gravity, is accompanied with constitutional symptoms like those attending phosphatic deposition, and originates in virtue of the same predisposing and exciting causes.

The diet of subjects discharging phosphatic urine is a point of primary consequence. Dr. Prout's experience proclaims loudly the superiority of nutritious *animal* food; M. Rayer's, as we stated on a former occasion, is to the same effect: the question may therefore be considered to be decided. But respecting the use of acids, whether medicinal or in the form of ascescent wines or fruits, we do not find the same harmony of opinion. Dr. Prout, as is well known, recommends the use of vegetable and of mineral acids; and when wine has been an ordinary article of consumption, Rhenish, Moselle, or Bucellas wines. Yet we must affirm that the aggregate of unprejudiced experience is opposed to the doctrine implied by such recommendation. Rayer well remarks, that acids rarely produce the desired effect on the urine, and that when their exhibition is persevered in for any length of time, they derange the digestive function, the free exercise of which is so essential a point to preserve so long as the progress of the malady itself will permit. We believe that Sherry or Madeira, of good quality, is better adapted for these patients than the class of wines spoken of by Dr. Prout. But we agree to the full with our author in the statement, of which, in our minds, it is impossible to exaggerate the importance that "absence from care, the exhilirating air of the country and such occupations (in moderation) as are consistent with the patient's peculiar condition, will perhaps, *more than anything else*, contribute to the cure; particularly in those slighter and induced cases, in which the affection is not complicated with local injury." Upon the value and mode of exhibition of sedatives and tonics we find nothing new. To the use of the shower-bath, especially of sea-water, as recommended here, we are accustomed to offer no objection, provided at first the trial of this powerful agent be very cautiously conducted. Dr. Prout, admitting the deposition of the mixed phosphates to be rare as an idiopathic affection, adduces three alleged examples of the fact in the form of cases. But instead of proving the point they are designed to illustrate, so far as the meagerness of detail and want of precision in the narrative permit us to judge, the unnatural abundance of those salts was in all three induced by organic disease of the urinary passages. It is a melancholy fact that the genius of many of our medical writers apparently induces them to despise the task of case collecting; of this we are certain that, judging from results, it incapacitates too many among them for entering upon this fundamental part, without which all others are as mere dross, of the investigation of disease with a view to the establishment of truth.

Some observations upon the phenomena attending the transition of different diatheses to the phosphatic close the present division of the work. One of the first changes usually detected by our author during the conversion of the oxalic into the phosphatic diathesis, was the secretion of an excess of (carbonate of) lime; he observed too that as the quantity of lime becomes greater, the proportion of the oxalic acid is decreased, while that of the phosphoric is increased, until at length phosphate of lime is deposited in nearly a pure state. The urine during these changes is frequently observed to let fall the triple phosphate also. The first circumstances denoting a change from the lithic to the phosphatic diathesis are the paleness and sometimes the increased quantity of the urine. There is also a great tendency to precipitation of pale amorphous sediments, generally with more or less of the phosphates. Though ascescent when voided, an irridescent pellicle of phosphates soon forms on the surface. On account of the rarity of the cystic oxide, Dr. Prout has had few opportunities of observing its transition into the phosphates.

We have now presented the reader with a tolerably full account of the contents of Dr. Prout's first book; the second hardly calls for equally complete notice, as it embraces topics both more familiar to practitioners and more familiarly treated by the author. Here are examined "mechanical diseases, comprehending the description and treatment of diseases arising from obvious lesions of the kidney and bladder; and particularly from the presence of concretions in those organs." The book is divided into seven chapters: three of these referring particularly to the growth and symptoms of calculi in the kidneys and bladder, and the modes of removing them, with remarks on the operations of lithotomy and lithotrity, we defer noticing for the present; of the others we shall limit ourselves to a very condensed abstract.

Dr. Prout commences his brief history of acute idiopathic nephritis with an affirmation that it is, at least in this country, a very rare disease. We have in another volume of this journal stated our reasons for not acceding to this notion, and find no motive in the pages before us for swerving from our then recorded impression. Dr. Prout may have "only seen two or three well-marked instances of the disease," and may not "be able to speak with *much precision* of more than one case," but these facts may be otherwise accounted for than by the presumed rarity of the occurrence. But admitting that such rarity exists, and that consequently little or nothing is generally known of the anatomical characters of nephritis, it surely was incumbent upon Dr. Prout accurately to describe the condition of the organ, when good fortune furnished him with the opportunity of doing so. And yet Dr. Prout can only speak with "much precision" of one of these cases. And what is the description given of this? A minute exposition of the condition of the arterial and venous vessels, of the colour, of the size, of the weight, of the consistence of the organ, of its fibrous, subfibrous, and mucous membranes, of the absence or presence of pus, of plastic lymph, or of gravel? No such thing; the pathological world are at once given the credit of perfect acquaintance with what but a moment before they are declared to have scarcely ever seen (and of which decidedly there is no good description in the English language); and instead of an account which might enlighten general ignorance, we find a simple assertion of the fact: we are simply told that the "kidneys were both much enlarged, and in a most intense state of inflammation throughout their whole substance." It is unnecessary to follow our author in his description of the disease, as confessedly it is not derived from experience, and falls consequently far short of the information already laid in different forms before the profession. We feel ourselves called upon however to notice some general statements, which to us appear to involve practical error. It is affirmed, for instance, that "what may be and is usually called chronic nephritis, is most commonly connected with a gouty diathesis and the formation of lithic-acid gravel or concretions." On the contrary, the researches of M. Rayer seem to prove that chronic nephritis is frequently independent of such diathesis, and is, when so independent, most commonly, as already mentioned, productive of phosphatic gravel. Speaking of rheumatic nephritis, Dr. Prout states "that the nearest approaches to what he has considered this affection have occurred in some of the milder cases of anasarca, accompanied by serous urine and produced by exposure to cold. In such cases the anasarcous swellings are often tender to the touch, and shift about without reference to the laws of gravity, very like rheumatic œdema." Here our pathologist becomes a mere symptomatic nosologist, in forming species from supposed differences in symptoms. M. Rayer has formally admitted a rheumatic nephritis, but upon the more judicious ground of his having detected a difference in the anatomical characters of the disease, when developed in rheumatic subjects. But Dr. Prout thinks meanly of such evidence as this, and prefers indulging in hypothesis as follows: "even acute anasarca itself might not be unreasonably referred to the universal inflammation of the same tissues which are usually the seat of common rheumatism."

The author alludes to the disease described by Rayer under the name of pyelitis—inflammation of the mucous membrane of the pelvis and calices,—but seems inclined to deprive the French observer of the merit of originality, by affirming that the "existence of the disease has long been suspected or rather known;" an affirmation which, as we are satisfied, would not stand before an enquiry into the evidence of the fact. Nor are the symptoms correctly stated: "the affection is accompanied by increased discharge of mucus and epithelium," says Dr. Prout; but he should have added, to do justice to Rayer whom he avowedly follows, that pus is poured out in more or less abundance. The French observer in truth lays great stress upon the alleged fact, that in cases of lumbar tumour and coexisting purulent discharge with the urine, the disease is really that now referred to, and not, as has been habitually supposed, chronic suppurative inflammation of the renal substance itself.

Dr. Prout supplies, in an enquiry into the diagnostic value of "pains in the back," some useful information as to the mode of distinguishing the different conditions of the kidneys to which such pains may be owing. The cases put by him all imply that the kidney is really the *seat* of the disease; its *nature* therefore is the only point to be established: as far as they go, Dr. Prout's observations may be referred to as of very great practical value; they are unfortunately too long for extraction.

As respects the treatment of nephritis, the author recommends venesection, the warm bath, and the exhibition of active doses of calomel conjoined with opium, henbane, and colchicum, &c.; (in gouty subjects mustard poultices to the feet), and when the disease has begun to yield under these measures, blisters; emollient clysters with or without opium should be administered, and strict antiphlogistic regimen insisted on.

In proceeding to discuss the subject of vesical diseases, Dr. Prout promises not to infringe on the province of the surgeon, and indeed there is no reason why he should, for assuredly their general pathology, diagnosis, and medical treatment, would in themselves supply abundant materials for an *ex professo* treatise. In the account of chronic inflammation of the mucous membrane of the bladder, cystorrhœa or catarrhus vesicæ, we find little to detain us. The ropy muciform matter which is now almost universally recognized both at home and abroad to be in reality pus, modified in property by the action of alkalies developed in the bladder, is still described by Dr. Prout as consisting of mucus. At the very outset of the complaint he has ascertained that the urine is acid, but the mucus itself always neutral, if not alkaline. As the disease advances however, the urine becomes alkaline; it has now a strong ammoniacal smell, and, as is well known, effervesces with an acid; according to the author there is "almost always in this case an excess of the carbonate of potash or of soda present; which are derived from the serum of the blood exuded from the ulcerated inner surface of the bladder." Towards the close, however, he has remarked that while the urine grows scanty and very high coloured, it occasionally reassumes an acid reaction, and the mucus or pus gradually diminish or almost disappear. The importance, as regards the prognosis of this change to acidity will become extreme, should the fact now announced meet with general confirmation. "I do not remember," says Dr. Prout, "to have ever seen a person recover when the urine has rather suddenly become acid, in long protracted and severe affections of the bladder usually accompanied by alkaline urine. After death the urine, though alkaline in the bladder, is often found acid in the kidney, provided that organ be not diseased."

Dr. Prout's account of irritable bladder is in some measure novel. The disease may depend either on functional or organic disease of the urinary organs, or upon nervous affections of a remote and constitutional origin. He first considers those cases in which the complaint depends upon functional derangements of the kidneys, and well points out that here the irritability directly depends on unnatural properties of the urine. Whenever that fluid varies in composition from its normal state, it becomes a source of *feeling*, a state which never arises in health except from repletion of that organ. In this mode the bladder may become occasionally irritable in persons of every age, but in young and healthy persons such irritation is only temporary. Dr. Prout appears to regard dyspepsia as the cause of this derangement, and conceives that in those whose assimilation is permanently bad, the tissues of the bladder may ultimately become organically diseased.

A much more fertile and more serious source of irritability of the bladder is disease of the kidney, a fact commonly known. Dr. Prout describes at some length, and as type of a class, an affection of those organs which he appears to consider especially connected with such derangement of the vesical functions. Respecting the anatomical nature of this renal affection we are left completely in the dark, unless the statement that "some of the affections formerly described as connected with anæmotrophy are so nearly related to, or so imperceptibly graduate into those now to be described, that it becomes impossible to draw the line between them," be accepted as conveying available information. Of the symptomatic and other characteristics of the disease as here stated, the following abstract comprises the more important particulars. It is principally confined to early and middle life, connected with a composite cachexia "*strumous*," "*remote syphilitic*," and "*malignant;*" hence often connected with the oxalic-acid diathesis and anæmotrophy of the system, never with decided lithic-acid diathesis; it is usually very slow, almost imperceptible to the patient in its earlier progress. The urine is generally acid, of a pale greenish whey-like colour, opalescent, below 1020 in specific gravity, often serous, rarely bloody, sometimes deposits the phosphates when heated, rarely a grayish ash-coloured sediment of the lithate of ammonia on cooling, and becomes clear by standing. The desire to make water is frequent and urgent, the period varying from one to three hours, and the quantity from one to three ounces; micturition is attended with scalding pain along the urethra, particularly behind the scrotum. There is no mechanical obstruction to the discharge, and in the early stages perfect ease is enjoyed after micturition, until the patient is again called upon to empty the bladder. With the advance of the disease all the symptoms increase, the calls are more frequent and urgent, the unnatural properties of the urine become more marked, the patient grows weak, emaciated, and irritable, and more than ever under the influence of atmospheric changes; he complains of no pain in the loins, but if pressed will sometimes admit the existence of a dull aching sensation there; the pulse gradually becomes quick and feeble, sickness occurs after eating, the urine diminishes in quantity, the patient complains of nothing, becomes drowsy, and at length goes off in a comatose state. Occasionally, however, the fatal event is hastened by an accidental exposure to cold or otherwise. The affection occurs in females as well as in males, and is occasionally attended with an increased vascularity and tumefaction about the orifice of the urethra, which part appears to the patient the chief seat of her distress. As respects the condition of the organs, Dr. Prout presumes that the whole secreting apparatus of the kidney is more or less involved, and that the mucous membrane of these organs takes on a diseased action capable of being propagated to the rest of the mucous surface as far as the bladder, in which viscus it appears even to commence sometimes, the symptoms varying in intensity according as the fundus or neck are attacked. Dr. Prout can say but little about the nature of this lesion; it may, he fancies, "in common language be called inflammation, but to his mind it is rather a species of degeneration." We would willingly draw from this some definite inference as to the morbid changes in the disease described, but from such data any other than a hypothetical conclusion cannot by possibility be derived.

Having thus described what "may be considered as the fundamental character of organic affections of the bladder, originating in kidney disease;" the author briefly notices some of "those rarer complications," which are liable to produce an impression of the existence of calculus. Under this head, polypous excrescences, elongations of the mucous membrane, fungus hæmatodes, &c. are ranged. The former affections are said to be usually in themselves harmless, and to cause inconvenience by their mechanical effects alone, unless they be complicated with the cachexia spoken of; and it is further affirmed, that fungus hæmatodes is a "tumour rendered malignant by being complicated with that cachexia." The nature of the structure composing morbid growths appears, in Dr. Prout's estimation, to exercise no influence on the phenomena induced by their presence, a notion totally opposed to the doctrines of the sagest pathologists. Be this as it may, however, fungus hæmatodes of the bladder is well known to become frequently more or less encrusted with phosphate of lime, and under these circumstances to have not unfrequently deceived surgeons into the belief of the presence of an ordinary calculus. Upon the question of diagnosis in these cases, the observations of Dr. Prout will have thrown some light, should it prove as he alleges it to be, a constant fact that the red particles of the blood discharged in the earlier stages of "fungoid disease" have a remarkable and characteristic appearance, seeming to the eye larger than natural, so that when they have subsided to the bottom of the urine, they at first sight somewhat resemble grains of lithic-acid gravel, and like that substance may be distinctly seen, when the vessel is inclined on one side, to roll along the bottom.

In the irritable states of the bladder which are commonly considered to depend upon nervousness, Dr. Prout has discovered that the properties of the urine are decidedly changed. Allied to this subject is that of the changes, real or simulated, which take place in the urine and urinary organs of hysterical females. Dr. Prout is too experienced a practitioner not to have encountered many cases of the flagrant deception which this order of females delight to play off upon those around them; deceptions the extent, occasionally disgusting character, and pertinacity of which appear in some instances to be only intelligible by supposing them dictated by some low form of insanity. His cautions to the young practitioner called upon to investigate one of these most difficult cases are valuable.

The summing-up remarks upon the diagnosis of these different affections of the bladder are most practical and judicious; they are really clinical, and as such we very strongly recommend them to the study of our younger readers. The subject of treatment is also very ably and comprehensively handled, and we regret that the near exhaustion of our space obliges us to confine our particular notice to the management of the affection described by Dr. Prout himself. In the early stages "if there be anything like activity," cupping the loins or leeching the perinæum are advisable, and the citrate of ammonia with mild purgatives is to be given internally. When instead of activity there is quiescence, the *uva ursi*, the *lythrum salicaria*, the *pareira brava*, and even small doses of the chalybeates, as the *tinct. ferr. muriat.*, are sometimes useful. In the more advanced stages, when the urine has become decidedly *albuminous*, and with a tendency to alkalescence, the citrate of ammonia either alone or combined with the fluid extract of sarsaparilla should be administered. If the disease be quite passive, the *infusum diosmæ* with sarsaparilla or muriatic acid may be given; or the *tinct. benz. co.*, the infusion of wild carrot seed or of sassafras, prescribed in such weak doses as never to excite. An issue or seton may be applied over the kidneys: this is not however often advisable. In the last stages of the disease, sedatives are the practitioner's only refuge, and of these *hyoscyamus*, *muriate* or *meconate of morphia* and *conium* are the best.

We shall on a future occasion examine the contents of Dr. Prout's chapters on hæmaturia and incontinence of urine.

Our general estimate of Dr. Prout's treatise may be gathered from the analysis just concluded, and the strictures scattered through it. We acknowledge and have pride in bearing testimony to the high qualifications of our countryman in the branch of pathological enquiry based upon chemical facts; we recognize the comprehensive sagacity of his speculations, and have respect for the patient zeal with which he has toiled to erect upon these a stable system. But we fear the time for such systematizing has not yet come; and although all speculations on the subject are seductive in themselves, and doubly so when emanating from an individual of Dr. Prout's eminent skill in the department of chemical physiology, it cannot, we think, be denied that in the existing unformed and vacillating state of organic chemistry, they sin essentially in being established on a most unsound basis. Nor can we avoid entertaining some solicitude as to the results of their propagation, which to us appears likely to betray minds of inferior order into mere extravagances. For these, however, Dr. Prout is not fairly answerable; and should his doctrines—when the frail embryo science on which they are based, has reached healthy maturity—be recognized as true, he must almost take rank with those highest intelligences, whose energy has outrun the scientific apprehension of their times. But meanwhile Dr. Prout has neither done his doctrines, himself, nor his readers justice, in not explicitly stating the foundation for and manner of verifying (so far as he is acquainted with these himself) his presumed results. The day has passed never to return, when the authority of name could at will supply the place of demonstration.* Should Dr. Prout intend favouring the public with further volumes, he would do well to ponder on these admirable remarks of a natural philosopher, of an eminence not inferior to his own: "Il ne suffit donc pas de dire qu'on a vu telle chose. Ce n'est rien dire, si, en même temps, on n'indique comment on l'a vue; si on ne met pas ses lecteurs en état de juger de la manière dont les faits qu'on rapporte ont été observés."†

There are some defects in the manner of treating the subject, which can scarcely be passed over without comment. No illustrations are here drawn from micrography and microchemistry, which from their recent successful application have been shown to be indispensable in these investigations. On the inefficient and sometimes, as we think, positively erroneous notions on pathological anatomy put forward in the volume, we have already animadverted. The classification of diseases into functional and mechanical, in itself bad, is lost sight of totally in the examination of details. By what laxity of phraseology can simple idiopathic nephritis be termed a mechanical affection, as it is here made to appear from the manner in which it is classified? Will it be believed again, by those who have not read the essay, that the severest and most advanced forms of Bright's disease, of encephaloid infiltration and of total destruction of the renal tissues, actually figure therein as so many "functional diseases"? There is, in truth, a tendency in the whole tenor of the work to establish a symptomatic medicine, to dispossess lesions of their pre-eminence, and invest symptoms with the importance of causes. The very

* Dr. Prout excuses himself from the statement of proof by the "practical" character of his book; this is the worst species of extenuation, as we think, which he could have adopted. What is not proved may be admitted in a merely theoretical work, intended to exercise the mind of closet speculators, because it can then do no direct and actual harm; but to extend to practice modes of acting founded altogether upon undemonstrated fancies, may, and is likely to become mischievous positively and physically.

† Trembley, Histoire d'un Genre de Polype, &c.

title of the volume is an illustration of this; of "stomach diseases," except on the title-page, we in reality hear nothing: of certain forms of dyspepsia much is no doubt said, but of the organic causes and essence of these not a word is breathed.

But we have done with this most irksome duty of exposing what we believe to be errors of no mean importance. We can most unaffectedly assure Dr. Prout, that it would have been infinitely more grateful to our feelings to lavish unmixed praise; but this we could not do. Were we to lend our voice to the establishment of doctrines such as those we have just taken leave of, we should break the vows we made on assuming the critical chair, to suffer no dogma which, in our conscience and to the best of our judgment, we believed to be wrong, to pass unscathed through our hands.

---

